# Efficacy and Safety of PreserFlo MicroShunt Implantation and Its Effects on Intraocular Inflammation through Laser Flare Photometry

**DOI:** 10.1155/2024/2447721

**Published:** 2024-07-23

**Authors:** Carlo Cagini, Niccolò Boni, Tommaso Bonifazi, Daniela Fruttini, Francesco Della Lena

**Affiliations:** University of Perugia School of Medicine and Surgery Department of Medicine and Surgery Section of Ophthalmology Ospedale S. Maria della Misericordia, Perugia 06156, Italy

## Abstract

**Purpose:**

The primary objective of this study is to evaluate the efficacy and safety profile of PreserFlo MicroShunt implantation in the medium- to long-term follow-up of patients with open-angle glaucoma. The secondary objective is to analyze laser flare meter (LFM) values before and after PreserFlo MicroShunt implantation.

**Methods:**

This prospective, observational, longitudinal single-center study included a total of 62 eyes from 54 patients. A subgroup of 27 eyes (26 patients) reached the 12-month follow-up. Success was defined based on three criteria: criterion A: IOP ≤21 mmHg and ≥20% reduction; criterion B: IOP ≤15 mmHg and ≥25% reduction; and criterion C: IOP ≤12 mmHg and ≥30% reduction. Success was further categorized as complete if achieved without IOP-lowering medications and qualified if achieved with medication administration. Other aspects evaluated included the number of IOP-lowering medications (baseline and postoperative), development of postoperative complications, 5-FU injections or implant revision, and LFM values.

**Results:**

The 12-month follow-up group (27 patients) was composed by 50% males and had a mean age of 75.54 ± 9.98 years. Success rates at 12 months were as follows: 78% for criterion A, 56% for criterion B, and 26% for criterion C. Complete success, as defined by criterion A, was achieved by 67% of the patients, 29% achieved qualified success, and one eye (4%) experienced failure. IOP decreased from 25.26 ± 1.67 mmHg at baseline to 14.81 ± 0.74 mmHg at 12 months. The number of medications decreased from 3.67 ± 1.30 at baseline to 0.48 ± 0.75 at 12 months. Reported complications were choroidal detachment (11%), hyphema (5%), and athalamia (flat anterior chamber) (2%) 13 eyes (48%) received 5-FU injections, while 7 eyes (26%) underwent implant revision. No significant increase in LFM values was observed. Eyes with a regular postoperative course and IOP ≤15 mmHg showed significantly lower LFM values than patients with unfavorable outcomes (IOP >15 mmHg, development of complications, 5-FU injection, or implant revision).

**Conclusions:**

PreserFlo MicroShunt showed a significant reduction in IOP and a decrease in the number of IOP-lowering medications. Complications occurred at a modest frequency. The implant provides a minimally invasive approach with no significant increases in LFM values postoperatively. Higher LFM values correlate with unfavorable postoperative outcomes.

## 1. Introduction

Glaucoma is a chronic progressive condition and is one of the leading causes of blindness worldwide [1]. Most therapeutic strategies focus on reducing intraocular pressure (IOP), which can be achieved through topical IOP-lowering medications, laser treatment, or surgical procedures [1].

In recent years, a series of new surgical techniques called Minimally Invasive Glaucoma Surgery (MIGS) have been developed to provide good efficacy in reducing IOP while lowering the rate of complications [2]. The PreserFlo® MicroShunt (Santen, Osaka, Japan) is a MIGS device that effectively reduces IOP by harnessing the principles of fluid dynamics, using an ab externo technique [3]. The shunt is made of an inert biocompatible material called polystyrene-b-isobutylene-b-styrene (SIBS), with a length of 8.5 mm and a lumen of 70 *μ*m. It is implanted through an ab externo approach, allowing the drainage of aqueous humor from the anterior chamber to a posterior bleb formed under the Tenon's capsule [3, 4].

The main reason for the failure of the PreserFlo MicroShunt is fibrosis and scarring of the bleb and the resulting increase in IOP due to mechanical obstruction of aqueous humor filtration. To address this, clinical practice has introduced tools to limit fibrosis, such as antimetabolites, which are now routinely used (5-fluorouracil and mitomycin C) [5, 6].

This study aims to demonstrate the efficacy and safety of the PreserFlo MicroShunt implant in patients with open-angle glaucoma, through a 1-, 3-, 6-, and 12-month follow-up evaluation of intraocular pressure (IOP), IOP-lowering medications, complications encountered, and the need for additional interventions such as revision surgeries and 5-fluorouracil injections.

To further support the minimally invasive nature of the intervention and attempt to identify signs of subclinical inflammation that may increase the risk of bleb scarring, a subgroup of patients underwent laser flare meter (LFM) measurements during follow-up. LFM is an instrumental examination that uses the principle of the Tyndall effect to obtain a quantitative measurement of protein levels in the anterior chamber of the eye, allowing for an objective numerical assessment of ocular inflammation and the integrity of the blood-aqueous barrier. It is a noninvasive, cost-effective, and repeatable examination already widely used for various ocular conditions [7]. The value obtained with the laser flare meter could be a tool to predict therapeutic failure early on, even before clinically evident pressure elevation, as it can identify eyes with a higher degree of inflammation and, therefore, a higher risk of fibrosis and scarring.

Studies applying LFM to glaucoma surgery are scarce and present heterogeneous results, and to our knowledge, no study has systematically applied LFM measurements to patients operated with the PreserFlo MicroShunt.

## 2. Materials and Methods

### 2.1. Study Design

The current study is a prospective, observational, longitudinal, monocentric study, involving patients who underwent PreserFlo MicroShunt implantation from February 2022 to April 2023 at the Ophthalmology Section of Santa Maria della Misericordia Hospital in Perugia, Italy.

A total of 68 eyes underwent PreserFlo MicroShunt implantation during this period. Postoperative data were available for 62 eyes at 1 month, 59 eyes at 3 month, 48 eyes at 6 months, and 27 eyes at 12 months.

The following exclusion criteria were set:

- Age <45 years old
- Pregnancy or nursing
- Refusal to participate in the study

Each patient included in the study underwent a comprehensive baseline assessment in which the following data were collected: age, sex, ethnicity, best-corrected visual acuity (BCVA), intraocular pressure (IOP) measurement by means of Goldmann applanation tonometry, number and class of IOP-lowering medications, glaucoma subtype, and any previous ocular surgical procedures.

During the follow-up period, the following variables were collected: IOP; number and class of IOP-lowering medications; intraoperative and postoperative complications; eventual need for subsequent 5-fluorouracil injection, surgical revision, or any other surgical interventions performed to counteract pressure increase.

Data were collected during visits conducted at the following time points:

- Baseline visit
- Day 1 visit
- Week 1 visit
- Month 1 visit
- Month 3 visit
- Month 6 visit
- Month 12 visit

If a patient had more frequent visits, the one closest to the selected time point was considered.

Success was stratified by arbitrary definition of three criteria:

- Criterion A: IOP ≤21 mmHg and ≥20% reduction from baseline
- Criterion B: IOP ≤15 mmHg and ≥25% reduction from baseline
- Criterion C: IOP ≤12 mmHg and ≥30% reduction

Success was further divided as “complete” if the requirements for each criterion success were met without the need for IOP-lowering medications and “qualified” if achieved with the use of medications. Failure was defined by the finding of an IOP >21 mmHg on two consecutive visits or by need to perform a new PreserFlo implant or a new glaucoma surgery intervention.

Time intervals of the visits, success criterions, and the definition of failure were chosen in accordance with the World Glaucoma Association guidelines on designing and publishing studies glaucoma surgery [8].

Additionally, a subgroup of 23 eyes (21 patients) underwent anterior chamber flare assessment using laser flare meter (LFM). The examination was performed at baseline, at week 1, at month 1, and at month 3. In this subgroup, all patients had a prostaglandin analogue (PGA) among their medications at baseline, so the influence of the possible pro-inflammatory effect caused by PGAs was neglectable.

The secondary objective of the study was to evaluate whether intraocular flare values in the postoperative period were correlated with therapeutic success. For this purpose, the group of 23 eyes was divided into 2 subgroups:

- Group A: this subgroup included eyes (*n* = 12) that had IOP ≤15 mmHg at 3 months and did not develop unfavorable outcomes (no complications, no re-introduction of IOP-lowering medications, no 5-FU injections, and no surgical revisions or secondary filtering surgery)
- Group B: this subgroup included eyes (*n* = 11) that had IOP >15 mmHg at 3 months or developed unfavorable outcomes (postoperative complications, need for re-introduction of IOP-lowering medications, need for 5-FU injections, surgical bleb revision, or secondary filtering surgery)

**Figure 1:**
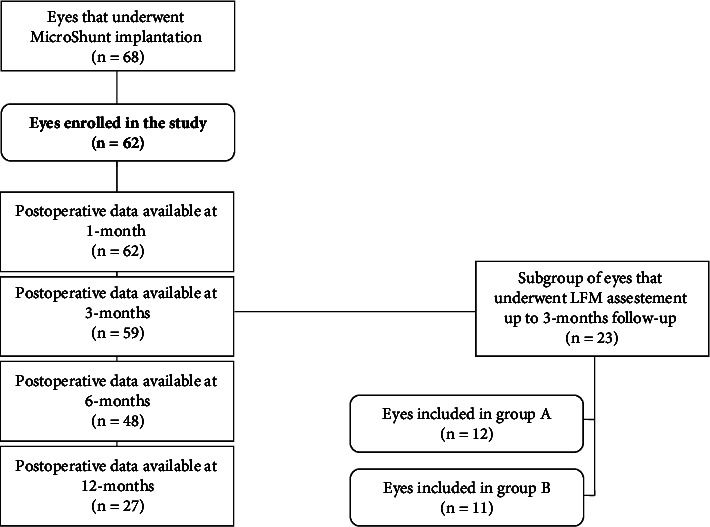
Flowchart showing the composition of the main group of eyes who underwent PreserFlo MicroShunt implantation in our center and the various subgroups into which it was divided to perform the statistical analyses.

A flowchart depicting the composition of the groups in our study is shown in Figure 1.

### 2.2. Surgical Procedure

MicroShunt implantation was performed in all patients by the same expert surgeon (C.C.) under topical anesthesia following the same procedure. A traction suture was applied to the clear cornea, and a conjunctival peritomy was performed at the limbus. The Tenon capsule was dissected from the episclera by means of blunt-tipped scissors to obtain a sub-Tenonian pocket. Three sponges soaked with mitomycin C (MMC) 0.2 mg/mL were then inserted inside the pocket for 3 minutes, and then removed with subsequent irrigation with saline solution to wash out any MMC excess. In case of abundant bleeding, diathermy was applied to the scleral vessels for cauterization. The sclera was marked at a point 3 mm from the limbus and a scleral incision was created using a 1-mm blade. A 25-gauge needle was then inserted inside the incision to create a tunnel reaching inside the anterior chamber through the iridocorneal angle. PreserFlo MicroShunt was inserted into the tunnel until 2 mm of the tip were visible in the anterior chamber. The fins of the MicroShunt locked into the scleral pocket to avoid migration. Aqueous flow was detected from the distal end of the shunt, and then the tail fins of the device were tucked within the scleral tunnel. Limbal sutures of the conjunctiva and Tenon capsule were performed and the traction suture was removed.

No intraoperative complications were reported.

### 2.3. Postoperative Treatment

All patients suspended their topical IOP-lowering treatment in the operated eye immediately after surgery. They also suspended oral acetazolamide if taken. Postoperative topical treatment composed of

- Moxifloxacin 0.5% eye drops, 4 times a day for 7 days, and then suspended
- Dexamethasone sodium phosphate 0.15% eye drops, 6 times a day for the first month, and then slowly tapered within 6 months until complete suspension

Postoperative treatment was not modified if the patient underwent 5-FU injection; however, it was started anew in the event of a surgical bleb revision (moxifloxacin 4 times a day for a week and dexamethasone 6 times a day).

### 2.4. Subconjunctival 5-Fluorouracil (5-FU) Injection

A subconjunctival injection of 5-fluorouracil (5-FU) was administered if the reduction in IOP failed to meet the desired target. In this case, the eye was disinfected with povidone-iodine 5% and topical anesthesia was applied. While the patient was asked to look down, 0.1 ml of 5-FU 5% were injected with a 30-G needle inserted at the margin of the bleb. The eye surface was then thoroughly rinsed with salt solution to wash out any 5-FU excess. No medications were given post-injection.

### 2.5. Surgical Bleb Revision

If IOP remained elevated despite 5-FU injections, a surgical revision of the bleb was performed by the same expert surgeon (C.C.). The conjunctiva of the bleb area was re-opened and the limbus with Tenon capsule dissected from the episcleral below, the terminal of the PreserFlo MicroShunt was exposed with mechanical lysis of any tissue adhesion. Mitomycin C was applied with 3 sponges left in place under the conjunctival pocket for 3 minutes, followed by rinsing of the site with balanced salt solution. The MicroShunt was primed and the conjunctiva and Tenon capsule were sutured again at the limbus.

### 2.6. Data Collection

IOP measurement was performed using a calibrated Goldmann applanation tonometer (Haag-Streit, Köniz, Switzerland).

Laser flare photometry (LFP) was assessed using a calibrated Kowa FM-700 Laser Flare Meter (Kowa Company Ltd, Nagoya Japan). In each visit, 10 LFP measurements were taken, and the highest and lowest readings were discarded. The mean value of the residual 8 measurements was then registered as the LFP value for that visit.

The number of IOP-lowering medications was calculated by adding the number of active principles in each patient's medical treatment, thus considering fixed combinations as separate medications. The categories of active principles considered as separate were prostaglandin analogues, beta-blockers, topical carbonic anhydrase inhibitors, alpha-2 agonists, and oral acetazolamide.

### 2.7. Statistical Analysis

The quantitative data collected are presented with the mean and standard deviation. For qualitative variables, the data are shown in tables as percentages. In some cases, the data are represented graphically.

To determine if the quantitative data follow a normal distribution, the Kolmogorov–Smirnov test has been used.

For the analysis of longitudinal data, repeated measures analysis of variance (ANOVA) or the nonparametric Friedman test has been employed, depending on the data distribution. Contrasts between means are evaluated using the Bonferroni test.

The significance level is set at *p* < 0.05.

## 3. Results

Clinical data and results used in this research have been deposited in the Open Science Framework repository [9].

### 3.1. Demographics

The composition of the groups of patients at various follow-up times is illustrated in Table 1.

### 3.2. Intraocular Pressure

Table 2 shows the mean IOP values at baseline and at every postoperative visit for each of the follow-up groups. A variance analysis was then performed using the repeated measures ANOVA test to compare the preoperative IOP values with those of the postoperative period, with a confidence interval of 95% and a significance level of 5% (*p* < 0.05). The analysis revealed a significant IOP reduction from baseline in each postoperative visit (*p* < 0.0001) in every group. Similar results were obtained through the Friedman rank test: in fact, mean IOP values were significantly lower in each postoperative visit than the one measured at baseline (*p* < 0.05) for every group.

At 6 months postoperatively, repeated measures ANOVA showed that IOP values at day 1, week 1, and month 1 were significantly lower than those measured at 6 months (*p* < 0.0001). However, there were no significant differences between mean IOP measured at 6 months and IOP measured at 1 and 3 months (*p*=0.753 and *p*=1.000, respectively). This was confirmed by a rank analysis with the Friedman test, which showed that IOP values at month 3 and month 6 did not differ significantly between them (*p*=1.000), while the difference in mean IOP between month 1 and month 6 was significant (*p* < 0.05).

At 12 months, the results obtained through the repeated measures ANOVA depict a similar scenario. In fact, IOP values at day 1, week 1, and month 1 were significantly lower than those measured at 12 months (*p* < 0.0001). IOP values did not differ significantly between 6 months and 12 months (*p*=1.000). The rank analysis with the Friedman test yielded similar results, as there were no significant differences in IOP values between month 6 and month 12 (*p*=1.000).

**Figure 2:**
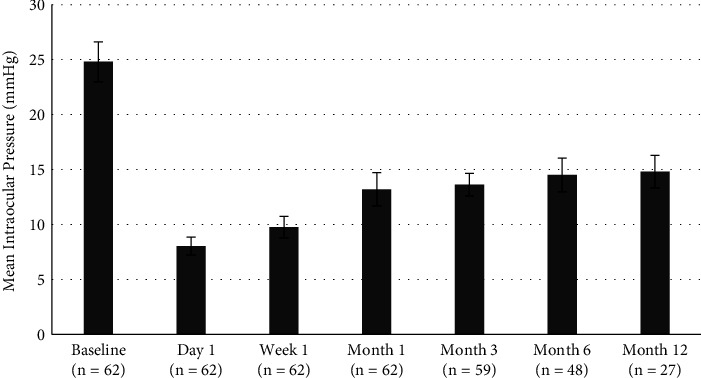
Graph showing mean IOP values during visits up to month 12. The number of patients at each follow-up visit is reported in brackets. The error bars represent the ±2SD of the values.

The mean IOP values at each visit during the 12-month follow-up are shown in Figure 2.

### 3.3. IOP-Lowering Medications

Table 3 shows the medications taken preoperatively and postoperatively in each follow-up group. We observed a reduction in the number of medications taken by the patients in each of the follow-up groups. The reduction from baseline was statistically significant in each of the follow-up groups according to the Wilcoxon test (*p* < 0.0001). In the group that reached 12 months of follow-up, the number of IOP-lowering medications decreased from 3.67 ± 1.30 at baseline to 0.48 ± 0.75 at 12 months postoperatively. Eighteen out of 27 eyes (67%) were medication-free at 12 months.

### 3.4. Complications and Additional Procedures

No intraoperative complications occurred, while postoperative complications were reported in a minority of cases. No signs of clinical inflammation (such as anterior chamber cells and flare o fibrin) were detected in any of the eyes at any follow-up visit after surgery.

Reported complications were choroidal detachment (11%), hyphema (5%), and athalamia (flat anterior chamber) (2%), all of which developed within the first month after intervention and resolved spontaneously within a week without the need of further intervention. In the only case of athalamia, the patient had grade 1 iridocorneal touch according to Spaeth's grading, that is, peripheral iridocorneal contact without corneal touching of the lens or IOL [10]. 5-FU injection was administered in 48% of the eyes, while 26% underwent revision surgery; only one eye experienced failure and underwent a new additional filtering surgery (namely, a new PreserFlo implantation in another quadrant). 5-FU injections were performed after 1 month from the intervention if the postoperative IOP increased over the clinical desired IOP target for that specific patient, and we performed a surgical bleb revision if the IOP remained elevated despite the 5-FU injections.

### 3.5. Criteria-Based Success Analysis in the 12-Month Follow-Up Group

In the group of patients with a 12-month follow-up, out of a total of 27 eyes, 21 (78%) met the success criteria, further stratified as follows:

- 21 (78%) met success criterion A (IOP ≤21 mmHg and IOP reduction ≥20% from baseline)
- 15 (56%) met success criterion B (IOP ≤15 mmHg and reduction ≥25%)
- 7 (26%) met success criterion C (IOP ≤12 mmHg and reduction ≥30%)

Out of the 21 eyes who reached success criterion A, 8 eyes (30%) required the re-introduction of IOP-lowering treatment, thus achieving qualified success, while the remaining 18 (48%) achieved complete success.

Despite the relatively small sample, IOP reduction at 12 months compared to baseline was statistically significant, decreasing from 25.26 ± 1.67 mmHg at baseline to 14.81 ± 0.74 mmHg.

Only one patient presented an IOP >21 mmHg in two consecutive visits (4%), thus defining the failure criterion. For this reason it was subsequently subjected to a new implant in the same eye.

**Figure 3:**
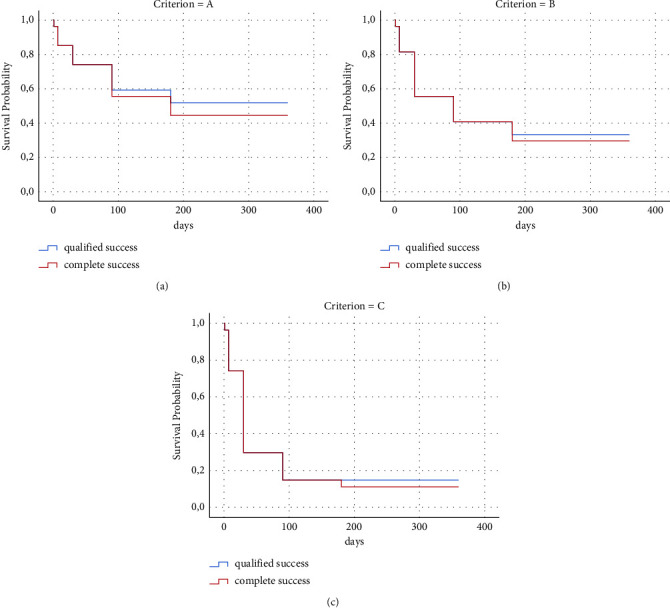
Graphs showing Kaplan-Meier curves for qualified and complete success according to study criteria (a) IOP ≤ 21 mmHg and reduction ≥20% from baseline, (b) IOP ≤15 mmHg and reduction ≥25% from baseline, and (c) IOP ≤ 12 mmHg and reduction ≥30% from baseline.

Figure 3 illustrates the Kaplan–Meier success rates as a function of the various success criteria for the group of eyes at 12-months follow-up.

### 3.6. 3-Month Follow-Up with Laser Flare Meter (LFM)

In the group of 23 eyes that underwent LFM assessment, mean preoperative LFM value was 24.00 ± 4.77 ph/ms, which slightly increased to 28.79 ± 4.28 ph/ms at week 1 and then decreased again to 17.21 ± 2.96 ph/ms at month 2 and to 13.84 ± 1.37 ph/ms at month 3.

However, statistical longitudinal analysis (repeated measures ANOVA) of the measurements indicated that there was no significant difference in LFM values during any of the follow-up visits (*p*=1.000).

Preoperative flare values were also comparable to those at 1 month (*p*=1.000) and 3 months of follow-up (*p*=0.096). There was also a significant reduction in flare from week 1 to month 1 (*p*=0.011), which was maintained until month 3 (*p*=0.004).

**Figure 4:**
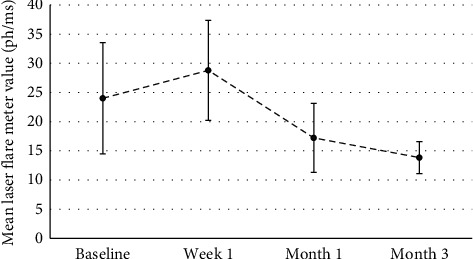
Graph showing mean LFM values during visits up to 3-month follow-up. The error bars represent the ±2SD of the values.

At the Friedman rank analysis, the results were similar: at week 1, the flare values were higher compared to the ones at 1 month and 3 months of follow-up. The values at preoperative and week 1 visits were comparable (see Table 4) (see Figure 4).

The LFM trend was then analyzed in the 2 subgroups that had different clinical outcomes:

- Group A: eyes (*n* = 12) with IOP ≤15 mmHg at 3 months and did not develop unfavorable outcomes (no complications, no re-introduction of IOP-lowering medications, no 5-FU injections, no surgical revisions, or secondary filtering surgery).
- Group B: eyes (*n* = 11) with IOP >15 mmHg at 3 months or developed unfavorable outcomes (postoperative complications, need for re-introduction of IOP-lowering medications, need for 5-FU injections, surgical bleb revision, or secondary filtering surgery).

In patients of group B that underwent 5-FU injections or surgical revisions before the 3-month visit (*n* = 5), the procedures were performed after the 1-month visit and before the 3-month visit in all cases, and the least time interval between the procedure execution and the 3-month LFM measurement was 3 weeks.

Friedman rank analysis was performed to separately evaluate the trend of LFM values for both groups over time. To directly compare the two groups, the Wilcoxon test was used.

The LFM average values showed different trends over time between the two groups. In Group A, the LFM values remained relatively stable from baseline up to 3 months of follow-up. In contrast, Group B values at 1 week were significantly higher than baseline and the subsequent controls at 1 month and 3 months.

**Figure 5:**
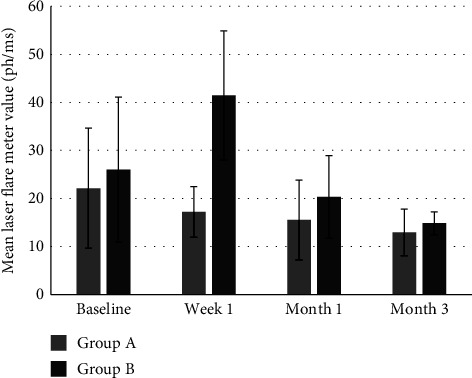
Graph showing the comparison of the LFM values between group A and group B up to 3-month follow-up. The error bars represent the ±2SD of the values.

To make a direct comparison between the two groups, the Wilcoxon test was performed too. Preoperative LFM values were comparable between the two groups (*p*=0.783), while at week 1 Group B values were significantly higher compared to Group A ones (17.18 ± 2.65 ph/ms in Group A, 41.46 ± 6.71 ph/ms in Group B) (*p*=0.008). LFM values then became comparable again between the two groups at month 1 (*p*=0.090) and month 3 (*p*=0.117) (see Table 5) (see Figure 5).

## 4. Discussion

Our study aimed to evaluate the efficacy and safety profile of PreserFlo MicroShunt implantation in the medium- to long-term follow-up of patients with open-angle glaucoma in our center. Our data seem to suggest that the PreserFlo MicroShunt demonstrates a good efficacy in reducing intraocular pressure, although it requires strict postoperative follow-up and minor surgical management to control possible complications and the risk of failure. We compared our results with other major studies on PreserFlo MicroShunt.

Schlenker et al. [11] evaluated the outcome of PreserFlo implantation in 164 eyes at 1-year follow-up. Success was defined if no 2 consecutive IOP measurements were above 17 mmHg and if >20% reduction from baseline IOP was achieved. As in our study, success was deemed qualified or complete if achieved with or without the use of glaucoma medications, respectively. The rates of complete and qualified success in their study (76.9% and 92.5%, respectively) were higher to ours (48% and 78%); however, it is difficult to make a comparison since the success criterions that were used in that study are different from ours. Furthermore, we experienced a higher rate of surgical revisions (26% vs 1.2%) as well as of other procedures (8.5% of needling in their case, 48% of 5-FU injections in ours). The cumulative incidence of complications, such as choroidal detachment (9.2%), are similar to ours (7% in the 1-year follow-up group).

In a multicenter study involving 81 PreserFlo patients, Beckers et al. [12] found a 74.1% rate of success (both with and without IOP-lowering medication) at year 1. This rate is very similar to ours (78%) and their success criterion for IOP (IOP ≤21 mmHg and reduction ≥20% from baseline) is the same as our criterion A.

Bhayani et al. [13] evaluated PreserFlo implantation in 100 eyes with a 12-month follow-up. Significantly fewer 5-FU injections and were performed in their experience (5%, compared to 48% in our center) at 12 months, as well as fewer bleb revision interventions (12% vs 27%). However, our center achieved similar success rates, both compete (48% vs 58%) and qualified (78% vs 74%). No hypotony-related complication was observed in their cohort.

We have two hypotheses regarding the higher rate of 5-FU injections and surgical revision in our study compared to others: on the one hand, some of the earlier cases we reported were among the first PreserFlo MicroShunts we implanted, so our experience on postoperative management was initially limited. On the other hand, we performed 5-FU injection every time the postoperative IOP increased over the clinical desired IOP target for that specific patient, so the IOP cut-off to perform it could be somewhat low, and that could explain the increased rate of antimetabolite injection. Moreover, we did not distinguish between needling and surgical bleb revision since in our center we do not perform needling procedures at the slit lamp, and we considered any bleb manipulation procedure as “surgical revision” since they are always performed in a surgery room. This could explain the higher rate of surgical revisions in our case.

Table 6 summarizes the results of some of the major PreserFlo MicroShunt studies compared to ours.

Detection and grading of anterior chamber inflammation during slit lamp examination relies on subjective evaluation of cells and flare; even though a standardization of inflammation grading has been established by the introduction of precise criteria by the Standardization of Uveitis Nomenclature (SUN) Working Group [15], this method is often prone to discrepancies due to intra- and inter-observer variability [16]. Laser flare photometry, by exploitation of Tyndall effect and laser light scattering, can obtain an objective, quantitative measurement of aqueous protein levels, thus allowing for a more precise quantification of the degree of blood-aqueous barrier disruption and ocular inflammation [7, 16]. It is a rapid an observer-independent tool that can assist in identifying subtle changes in aqueous composition and subclinical inflammation, in a stage when clinical detection of anterior chamber flare might be missed. As a matter of fact, SUN criteria do not include a 0.5 grade for flare, in opposition to cell grading where a 0.5 grade is present [17]. This is probably due to the fact that flare grading is merely based on the perception of “haziness” when observing anterior chamber details, and not on a more consistent parameter such as counting cells when grading anterior chamber cell presence. Laser flare photometry could therefore improve detection of early-stage inflammation when clinical flare or cells are not present at slit lamp examination.

Regarding the results obtained in the follow-up with the laser flare meter (LFM), the mean flare values in the postoperative period were comparable to the preoperative values in each visit up to 3 months of follow-up, and there were no significant increases in the immediate postoperative period (*p* > 0.05). This would cautiously suggest that PreserFlo MicroShunt implantation causes minimal trauma for the eye and the blood-aqueous barrier and does not result in a significant increase in intraocular inflammation. Additionally, no study in literature has yet systematically evaluated the level of ocular inflammation with LFM in patients who underwent PreserFlo MicroShunt implantation. Other studies, such as those by Tanito et al. [18], have followed patients operated with other types of MIGS (iStent and Microhook ab interno trabeculectomy), showing similar results as our center's, as non-significant increases in inflammation following the intervention were reported.

The level of intraocular inflammation affects the risk of fibrosis, scarring and device obstruction, thus reducing the rate success. Currently, the application of anti-fibrotic agents like mitomycin C and 5-FU is widely used in clinical practice [17, 19, 20]. Therefore, we decided to correlate the level of ocular inflammation measured by LFM with the therapeutic success achieved after the implantation of PreserFlo MicroShunt. Group A consisted of eyes that had an IOP ≤15 mmHg at 3 months and a normal postoperative follow-up, while group B included eyes that had an IOP >15 mmHg or an IOP below this value but experienced unfavorable outcomes (complications, re-introduction of IOP-lowering medications, 5-FU injections, revisions, or re-interventions). The group of eyes with an unfavorable follow-up showed significantly higher inflammation level at 1 week compared to the other group (41.46 ± 6.71 ph/ms vs 17.18 ± 2.65 ph/ms). In 5 Group B patients, potentially inflammatory procedures (5-FU injections and surgical bleb revisions) were performed before the 3-month visit but after the 1-month visit, and in no case the procedure was administered less than 3 weeks before the 3-month LFM measurement. While these interventions could lead to an increase in the inflammatory response and lead to an increase in LFM values, the effect on our results was reduced by this time interval. Moreover, if the procedures altered the flare values, we would expect an increase in LFM values at month 3 compared to month 1 in group B, which we did not observe. Therefore, while we cannot be certain that our additional procedures were not the cause of the increased LFM values in at least some of the patients in group B, we can affirm that they were not the cause of the statistically significant difference in LFM values between group A and group B observed at week 1.

Our single-center study was conducted in a real-life setting with consistent data collection, thus enhancing the reliability of our results and their representativeness of real clinical conditions. The use of laser flare photometry in glaucoma filtering surgery is an aspect rarely explored by current literature. Our results provide new insights into the role of subclinical inflammation in glaucoma filtering surgery and may stimulate new discussions in the field.

In conclusion, our results suggest that the level of ocular inflammation after PreserFlo MicroShunt implantation, measured by means of laser flare photometry, could be a key factor in the surgical outcome and may be an early indicator of unfavorable outcomes. This is particularly important considering that we have tools to counter inflammation (such as antimetabolites), and it could be valuable to have instruments like LFM that can predict who might benefit from them early on, even before significant increases in IOP are detected by Goldmann tonometry.

### 4.1. Limitations and Future Perspectives

It is essential to consider the limitations of our study.

Our study enrolled a relatively small number of patients both in the efficacy and safety analysis and in the LFM analysis. The results and the power of statistical significance can be affected by this factor, and we suggest that larger sample of patients should be enrolled in an LFM cohort in future studies. To the best of our knowledge, this is the first laser flare analysis performed on patients with PreserFlo MicroShunt, so our results should be intended as preliminary.

Intraocular inflammation can be affected both by complications and by postoperative procedures such as 5-FU injections or surgical revisions. Our study found a correlation between higher flare values and potential negative outcomes following PreserFlo MicroShunt implantation. While we have discussed postoperative inflammation and wound healing response as a possible explanation, this does not necessarily imply a direct cause-effect connection and higher flare values could be due to the complications themselves or to subsequent manipulation procedures such as 5-FU injections or bleb revisions.

There are various intra- and interindividual factors that make the measurements not always entirely predictable and reliable, such as the patient's immune response, glaucoma subtype, use of medications that interfere with the integrity of the ocular blood-aqueous barrier, and other unidentified factors. While all of the patients in our LFM subgroup were treated with PGAs at baseline, a more critical and complete investigation could be performed in future studies to further correlate preoperative use of PGAs to postoperative flare due to their potential pro-inflammatory side effects.

Endothelial cell (EC) evaluation is also an important aspect to consider when reporting complications pertaining to tube shunt insertion inside the anterior chamber. Our study did not include EC count and we did not experience any corneal decompensation nor any other complication due to EC damage caused by the device. However, we suggest that future studies on tube shunt should always include an EC count evaluation to detect changes over time.

## Figures and Tables

**Table 1:** Demographic analysis of the sample of patients and eyes included in the longitudinal analysis at various follow-up times (1-, 3-, 6-, and 12-month follow-up).

	Month 1	Month 3	Month 6	Month 12
No. eyes/patients	62/54	59/53	48/43	27/26
Gender				
Male, *n* (%)	29 (54%)	29 (55%)	22 (51%)	13 (50%)
Female, *n* (%)	25 (46%)	24 (45%)	21 (49%)	13 (50%)
Age, yrs (mean ± SD)	72.96 ± 11.14	72.64 ± 11.02	73.53 ± 10.68	75.54 ± 9.98
Glaucoma subtype, no. eyes (%)				
POAG	53 (85%)	52 (88%)	43 (90%)	26 (96%)
Pseudoexfoliative glaucoma	7 (11%)	5 (8%)	4 (8%)	1 (4%)
Neovascular glaucoma	1 (2%)	1 (2%)	1 (2%)	0 (0%)
Pigmentary glaucoma	1 (2%)	1 (2%)	0 (0%)	0 (0%)
Previous surgery, no. eyes (%)				
Cataract surgery	33 (53%)	31 (53%)	27 (56%)	13 (48%)
Keratoplasty	3 (5%)	3 (5%)	3 (6%)	1 (4%)
Vitrectomy	2 (3%)	2 (3%)	2 (4%)	1 (4%)
Xen implant	6 (10%)	6 (10%)	6 (13%)	2 (7%)
Trabeculectomy	1 (2%)	1 (2%)	1 (2%)	0 (0%)
Lens status, no. eyes (%)				
Phakic	29 (47%)	28 (47%)	21 (44%)	14 (52%)
Pseudophakic	33 (53%)	31 (53%)	27 (56%)	13 (48%)

**Table 2:** Mean IOP values up to 12 months of follow-up, with standard deviation (SD) and 95% confidence interval (CI).

Mean IOP ± SD [95% CI] (mmHg)				
Visit	1-month follow-up group (62 eyes)	3-month follow-up group (59 eyes)	6-month follow-up group (48 eyes)	12-month follow-up group (27 eyes)
Baseline	24.81 ± 0.90 [23.01–26.60]	24.95 ± 0.94 [23.07–26.83]	24.46 ± 1.06 [22.32–26.60]	25.26 ± 1.67 [21.83–28.69]
Day 1	8.05 ± 0.40 [7.25–8.84]	7.93 ± 0.40 [7.13–8.73]	7.98 ± 0.47 [7.03–8.93]	8.48 ± 0.75 [6.94–10.02]
Week 1	9.77 ± 0.49 [9.00–10.55]	9.78 ± 0.41 [8.96–10.60]	10.04 ± 0.46 [9.11–10.97]	10.81 ± 0.68 [9.43–12.20]
Month 1	13.21 ± 0.76 [11.68–14.74]	12.98 ± 0.79 [11.40–14.56]	13.00 ± 0.72 [11.55–14.45]	14.41 ± 0.78 [12.81–16.00]
Month 3	—	13.63 ± 0.51 [12.60–14.65]	13.50 ± 0.55 [12.39–14.61]	14.44 ± 0.72 [12.97–15.92]
Month 6	—	—	14.52 ± 0.76 [13.00–16.04]	15.04 ± 0.92 [13.14–16.94]
Month 12	—	—	—	14.81 ± 0.74 [13.29–16.34]
Repeated measures ANOVA	*p* < 0.0001	*p* < 0.0001	*p* < 0.0001	*p* < 0.0001
Friedman test	*p* < 0.05	*p* < 0.05	*p* < 0.05	*p* < 0.05

The reduction in IOP from baseline to each of the follow-up visit was statistically significant according to the repeated measures ANOVA and the Friedman test (*p* values are also reported).

**Table 3:** Number of IOP-lowering medications preoperatively and postoperatively.

	Month 1 (62 eyes)	Month 3 (59 eyes)	Month 6 (48 eyes)	Month 12 (27 eyes)
Number of eyes by number of preoperative medications				
0	1 (2%)	1 (2%)	1 (2%)	1 (4%)
1	3 (5%)	2 (3%)	1 (2%)	0 (0%)
2	12 (19%)	10 (17%)	9 (19%)	4 (15%)
3	17 (27%)	17 (29%)	10 (21%)	6 (22%)
4	12 (19%)	12 (20%)	12 (25%)	7 (26%)
5	17 (27%)	17 (29%)	15 (31%)	9 (33%)
Number of eyes by number of postoperative medications				
0	47 (76%)	47 (80%)	36 (75%)	18 (67%)
1	9 (15%)	7 (12%)	7 (15%)	5 (18%)
2	6 (10%)	5 (8%)	5 (10%)	4 (15%)
Mean number of preoperative medications ± SD	3.40 ± 1.30	3.49 ± 1.26	3.58 ± 1.29	3.67 ± 1.30
Mean number of postoperative medications ± SD	0.34 ± 0.65	0.29 ± 0.62	0.35 ± 0.67	0.48 ± 0.75

**Table 4:** LFM mean values up to 3 months of follow-up.

Visit	Mean LFM value ± SD (ph/ms)	Median (ph/ms)	95% CI (ph/ms)
Baseline	24.00 ± 4.77	16.30	14.11 to 33.89
Week 1	28.79 ± 4.28	19.30	19.92 to 37.66
Month 1	17.21 ± 2.96	11.00	11.67 to 23.96
Month 3	13.84 ± 1.37	11.70	11.00 to 16.69

Median and confidence interval (CI) are also reported.

**Table 5:** Comparison between mean LFM values over time in the group of eyes with a normal postoperative course (group A) compared to the group of eyes that developed unfavorable outcomes (group B).

Visit	Group A Mean LFM value ± SD (ph/ms)	Group B Mean LFM value ± SD (ph/ms)	*p* value (Wilcoxon test)
Baseline	22.13 ± 6.26	26.04 ± 7.53	0.783
Week 1	17.18 ± 2.65	41.46 ± 6.71	0.008^*∗*^
Month 1	15.53 ± 4.15	20.31 ± 4.30	0.090
Month 3	12.93 ± 2.43	14.85 ± 1.18	0.117

The difference in flare values between the 2 groups at week 1 was statistically significant according to the Wilcoxon test (^*∗*^).

**Table 6:** The main outcomes after PreserFlo microshunt implantation in different studies.

Study	Follow-up (months)	Eyes	Pre-IOP (mmHg)	Post-IOP (mmHg)	Qualified and complete success (%)	Pre-no medications	Post-no medications
Schlenker et al. [11]	12	164	21.4 ± NR	13.3 ± NR	92.5 and 76.9	3.4 ± NR	0.5 ± NR
Beckers et al. [12]	24	81	21.7 ± 3.4	14.1 ± 3.2	74.1	2.1 ± 1.3	0.5 ± 0.9
Bhayani et al. [13]	12	100	21.5 (19–28)	13 (11–16)	74 and 58	3 (2–3)	0 (0–0)
Ibarz Barberá et al. [14]	9–12	64	22.03 ± 6.38	12.92 ± 3.48	70.31 and 12.5	2.70 ± 0.72	0.19 ± 0.52
Our study	12	27	25.26 ± 1.67	14.81 ± 0.74	78 and 48	3.67 ± 1.30	0.48 ± 0.75

## Data Availability

The clinical data used to support the findings of this study and the statistical analysis performed have been deposited in the Open Science Framework repository (https://doi.org/10.17605/OSF.IO/GEQ8Z).
